# A 25 Mbps 15 ns Propagation Delay 150 kV/μs CMTI Configurable Dual-Channel Capacitive Digital Isolation Driver

**DOI:** 10.3390/mi15070811

**Published:** 2024-06-21

**Authors:** Yuan Zhao, Liang Wang, Zhifeng Chen, Boyang Li, Ronghua Zheng, Chengying Chen

**Affiliations:** 1 School of Opto-Electronic and Communication Engineering, Xiamen University of Technology, Xiamen 361024, China; bula121238@163.com (Y.Z.); hahahamm1232023@163.com (Z.C.); 2 School of Physical and Electronic, Henan University, Kaifeng 475000, China; doubleelectr@163.com; 3 Xiamen NewSound Technology Co., Ltd., Xiamen 361024, China; m.rd@newsound.cn (B.L.); m.hr@newsound.cn (R.Z.)

**Keywords:** capacitive isolation, half-bridge drive, on–off keying modulation, common-mode transient immunity

## Abstract

Electrical isolation devices are essential components for safeguarding the reliability of electronic systems under harsh conditions. Digital isolators are widely used in low-power circuits due to their high immunity to disturbances. In this paper, a capacitive digital isolator for high-efficiency power supply scenarios is proposed with a high common-mode transient immunity (CMTI) and high data transmission rate. The on–off keying (OOK) modulation technique is used to ensure a high speed and accurate signal transmission. A fully integrated high-voltage level-shift driver with an ns-scale delay is proposed for increasing the drive capacity. Post-simulation results in Cadence IC 6.1.7 with the standard 0.18 μm CMOS process show that the proposed architecture achieves a 25 Mbps data transmission rate and 15 ns typical propagation delay with output peak currents of 2 A/4 A, respectively. Meanwhile, a CMTI of more than 150 kV/μs is realized.

## 1. Introduction

Digital isolators protect systems and users from potentially hazardous voltages in high-voltage environments [1,2,3]. Digital isolators based on the CMOS process are widely used in various applications due to their small size and high reliability [4,5]. Digital isolators with common-mode transient immunity (CMTI) become basic devices in low-power applications. The development of digital isolators is directed towards two directions, reducing cost and optimizing performance [6]. Inductive coupling and capacitive coupling are two common forms of isolation in CMOS-based digital isolators.

Isolators based on inductive coupling utilize their self-inductive properties to realize long-distance data transfer [7]. However, transformers typically require significant space, and even require another mould for placement. The coil diameter of the transformer in [8] is as high as 230 μm, even requiring silicon-based post-processing techniques, which leads to increased costs. Pulse modulation technique is used in [9] to address the area and power consumption concerns. However, a GHz-range signal is generated, which affects noise immunity. Finally, the CMTI of [9] is limited to 25 kV/µs. A transformer isolator based on time multiplexing is presented by [10], which uses a 0.1 mm-thick isolation coefficient as the dielectric, but only achieved a 100 kV/µs CMTI. In contrast, capacitive isolators utilize SiO_2_ as the isolation dielectric material, whose field strength is about 400–500 Vrms /µm at room temperature. In a standard 0.18 μm CMOS process, a 5 µm thickness between Metal 1 and Metal 4 can theoretically achieve an isolation withstanding capacity of 2.5 kV. Compared to inductive isolation, capacitive isolation operates on electric fields alone and can be used without interference from the magnetic field of the surrounding MRI equipment. Additionally, the robustness under fast transient changes makes it more advantageous [11]. Due to the higher data rate, lower latency, and reduced cost, the capacitive isolator has gradually replaced the traditional magnetic coupler isolator in many applications.

The modulation methods of digital isolators can be broadly categorized into edge modulation and on–off keying (OOK) modulation. The way of encoding using the edge of the signal is easily affected by noise interference. In contrast, OOK modulation technology can significantly improve communication efficiency, and reduce transmission distance and transmission loss, while ensuring communication reliability.

Most current commercial isolation products have a narrow supply voltage range. For example, the capacitive digital isolator presented in [12] offers ultra-low power dissipation, but the typical application is a loop-powered field transmitter from 4 mA to 20 mA. An isolator that supports high transmission rates from DC to 50 Mbps per channel was proposed in [13], but the output current is only 4 mA. Moreover, most products have independent isolated channels, which makes it difficult to use them flexibly in different scenarios. In addition, the common-mode rejection (CMR) of many commercial products is limited to 100 kV/μs [14,15,16].

This paper proposes a dual-channel configurable capacitively coupled digital isolation driver based on OOK modulation. The transmitter (TX) die and receiver (RX) die are configured with a capacitive isolation barrier, and the two capacitors are connected back-to-back, which doubles the channel breakdown voltage and improves the isolation capability of the chip. Each channel is equipped with an independent shutdown pin. An external pin is designed to enable a programmable dead time function. With an input voltage range of 3.3 V to 5 V and an operating voltage of up to 20 V on the output side, it is suitable for connecting digital and analogue controllers. The proposed isolated driver architecture provides an up to 25 Mbps data rate and an up to 2 A/4 A output current with a typical propagation delay of 14.5 ns, while achieving an excellent CMTI capability of more than 150 kV/µs.

The organization of this paper is as follows: Section 2 describes the proposed digitally isolated driver architecture in detail, including the design of the transmitter TX, RX, and Driver sections. The post-emulation results are presented in Section 3, and conclusions are drawn in Section 4.

## 2. Materials and Methods

**Figure 1:**
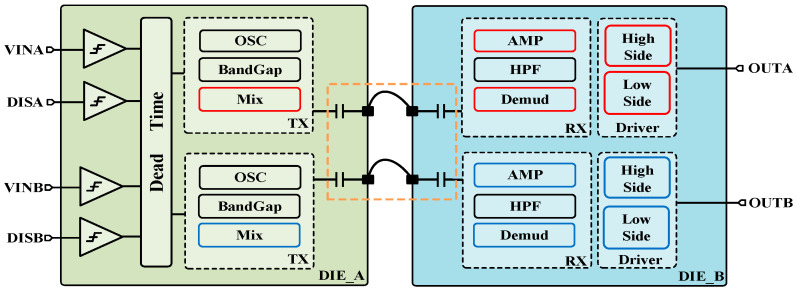
Overall architecture of the proposed digital isolated driver.

**Figure 2:**
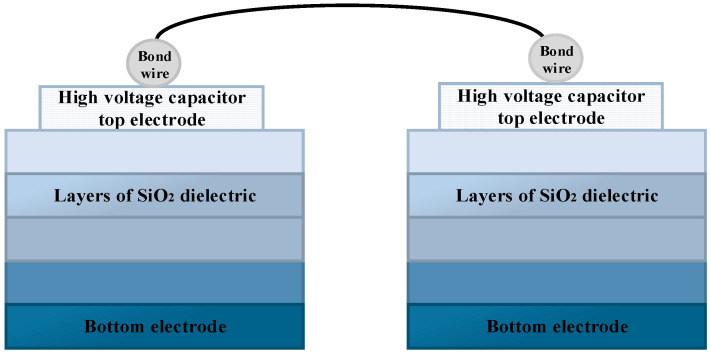
Cross-section of high-voltage isolation capacitor.

Figure 1 shows the overall architecture of the proposed digital isolated driver. The input side and output side are placed on separate substrates and packaged in a single package. Two metal–oxide–metal capacitors are used as isolation capacitors to match the fully differential transmission mode used in the TX section. The sectional schematic of the integrated isolation capacitor used is shown in Figure 2. The size of this isolation capacitor is determined by the area of the top and bottom pole plates and the distance between the plates. In 0.18 μm CMOS five-layer metal interconnect process, Metal 1 is used as the lower plate, while Metal 5 is used as the upper plate, and a silicon dioxide dielectric with a pitch of 4.8 μm is used as the isolation barrier, with a capacitor of 50 fF each. With two capacitors placed consecutively between TX and RX, a reinforced isolation level of 4.2 kVRMS can eventually be achieved.

The TX on input side include a Schmitt trigger for filtering, an internal oscillator (OSC) to generate the carrier signal, and a mixer to modulate the origin signal. The RX on the output side consists of a high-pass filter (HPF), a pre-amplifier, and a demodulator for accurately reconstructing the initial signal with low transmission delay. The output stage is designed as a fully integrated high-voltage level-shifted high-side and low-side driver, in order to minimize the passive device area of the isolator, ultimately providing sufficient drive capability with a 1.2 nF load connected.

The designed isolated driver can be configured as two signal-side drivers or a half-bridge driver with adjustable dead time. Two channels have independent ‘enable control’ pins. The chip operates normally when ports DISA and DISB are connected to a low level, and the outputs are pulled low when connected to a high level. The DT pin is used to control the dead time between two channels. When DT is high, channels operate independently and dead time control function is disabled. When DT is connected with a resistor to ground, the dead time between channels is determined by the magnitude of the external resistor.

### 2.1. Transmitter

**Figure 3:**
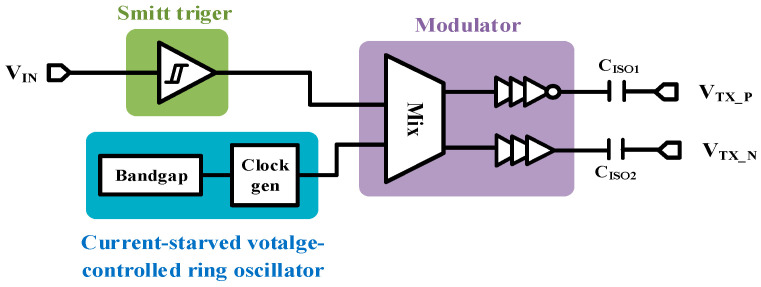
Block diagram of TX.

The overall block diagram of the TX is shown in Figure 3. As an important part of the transmitter, the built-in bandgap generates a high-resolution reference voltage that is less disturbed by environmental factors, enabling the whole system to work stably over a wide temperature range [17]. The input signal is first converted from analogue to digital by a Schmitt flip-flop, which shapes the signal and removes noise, enhancing the overall immunity of the circuit. As an implementation of ASK modulation, the analogue multiplier mixes the input signal with the carrier signal by using electronic switches to control the output of the oscillator and turn it off [18]. When the input is high, the output is a high-frequency carrier signal, and, when the input is low, the output is a low signal. The insensitivity of this modulation method to the data transmission rate results in high immunity.

The maximum data transmission rate of this design driver is 25 Mbps. According to Nyquist’s sampling theory, the output frequency of the oscillator should be at least twice the frequency of the input signal to avoid aliasing of the output signal [14]. In practice, to achieve higher transmission reliability and stronger immunity to disturbances, the input signal is often made to contain more than five oscillation cycles within the minimum pulse width. On the other hand, to maximize the rate of data transmission, the OSC should be kept oscillating at a high frequency. After considering the above, the oscillator frequency is designed at 200 MHz. We use current-starved voltage-controlled ring oscillator (CSVCRO) structure to reduce the influence of PVT variation [15,16]. A self-biasing circuit is used to control the bias current of the inverter, and the time of charging and discharging the load capacitor is controlled by fixing the bias current to minimize the frequency error caused by PVT variation. Oscillator with CSVCR structure exhibits good robustness, omits additional calibration steps, and simplifies the circuit topology, offering advantages in terms of area and cost savings.

**Figure 4:**
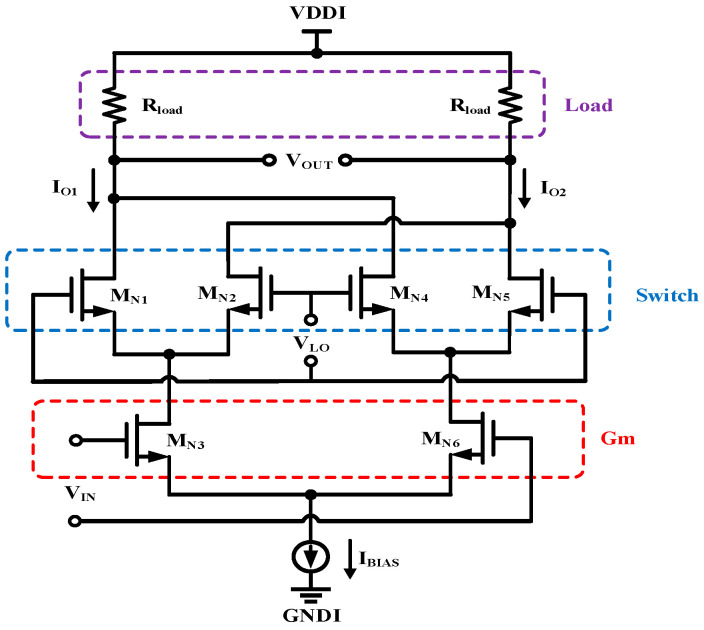
Double-balanced mixer Gilbert cell.

Figure 4 shows the architecture of the Gilbert double-balanced analogue multiplying mixer. The input signal *V*_IN_ is amplified in the Gm stage, frequency down-converted to a current signal in the switching stage, and then converted to a voltage signal by the output load stage. Compared to single-transistor or single-balanced mixers, the double-balanced mixer offers better isolation between its ports, and the currents of the upper two differential pairs are super-imposed in opposite phases, which cancels out the leakage of the input signal to the mediate frequency port. The conversion efficiency of the multiplying mixer depends only on the input current and current gain. When the input voltage changes, the Gilbert cell form a rapidly responding path to trigger the output.

**Figure 5:**
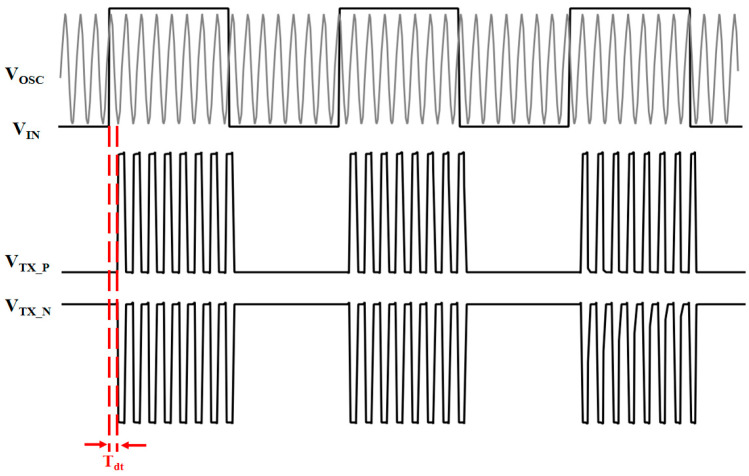
Diagram of main signal waveforms in TX.

Figure 5 shows the operating waveform diagram of the mixer, where Tdt is its propagation delay. The adopted form of OOK modulation minimizes the overall delay of the TX section.

### 2.2. Receiver

**Figure 6:**
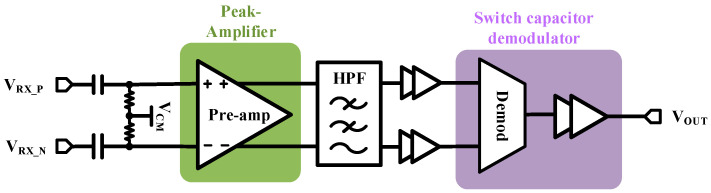
Block diagram of RX.

Figure 6 shows the proposed RX structure. The RX should be designed to take into account the DC operating point shift caused by common-mode transient events, and the high-frequency signal should be recovered from which it is attenuated by the isolation capacitors. The pre-amplifier is adopted to amplify the carrier waveform. In addition, it should maintain normal operation during the occurrence of voltage surges. The high-pass filter (HPF) removes the common-mode noise and reduces pre-amplifier offsets. The envelope detector (ED) is used for more efficient demodulation and reconstruction of the initial input signal.

**Figure 7:**
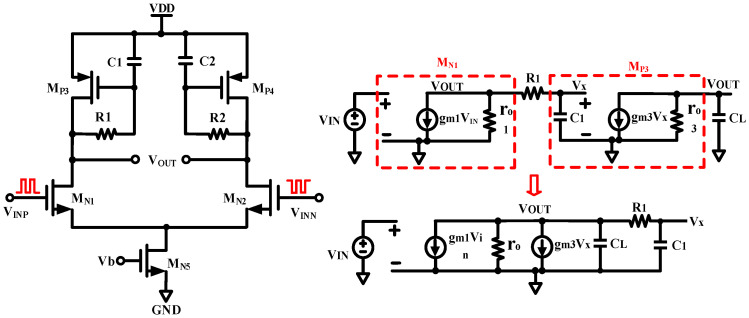
Peak amplifier diagram and small-signal model.

The peak gain pre-amplifier and its small-signal model are shown in Figure 7. A wide-bandwidth amplifier without high-impedance nodes is designed to achieve RF-level bandwidth. The fully differential form of dual-in-dual-out (DIDO) is used to suppress low-frequency common-mode signals effectively [19]. For low-frequency inputs, the single-side circuit can be equated to a common-source pole amplifier circuit with a PMOS diode connection as the load, and the gain can be calculated by Equation (1). Therefore, with proper size of M1 and M3, a gain of 1× (or slightly less than 1×) peak amplifier at low frequencies can be achieved.

(1) $$A_{V}=Gm\cdot Rout=g_{m1}\cdot \frac{1}{g_{m3}}=\frac{g_{m1}}{g_{m3}}=\frac{u_{n}c_{ox}{\left(w/l\right)}_{1}\left(V_{GS1}-V_{THPN}\right)}{u_{p}c_{ox}{\left(w/l\right)}_{3}\left(V_{GS3}-V_{THP}\right)}$$

For high-frequency inputs, *R*_1_ and *C*_1_ form a low-frequency filtering circuit. In AC small-signal model of the circuit, *C*_L_ represents the total capacitor from the output node to ground, and *r*_o_ is equal to *r*_o1_//*r*_o2_. Collation yields the small-signal gain *G*(s) of the peak amplifier as:

(2) $$G(s)=\frac{V_{OUT}}{V_{IN}}\left(s\right)=\frac{g_{m1}(1+sC_{1}R_{1})}{C_{L}C_{1}R_{1}s^{2}+(\frac{C_{1}R_{1}}{r_{o}}+C_{L}+C_{1})s+\frac{1}{r_{o}}+g_{m3}}$$

The peak response gain with bandpass effect can be achieved by selecting the RC value under certain conditions. It is worth noting that, if a narrower passband bandwidth is chosen for better filtering, although it can suppress low-frequency common-mode signals, its gain at the peak will be greatly reduced and the transient response is prone to distortion. Therefore, the choice of frequency bandwidth for the signal amplification region requires a trade-off.

**Figure 8:**
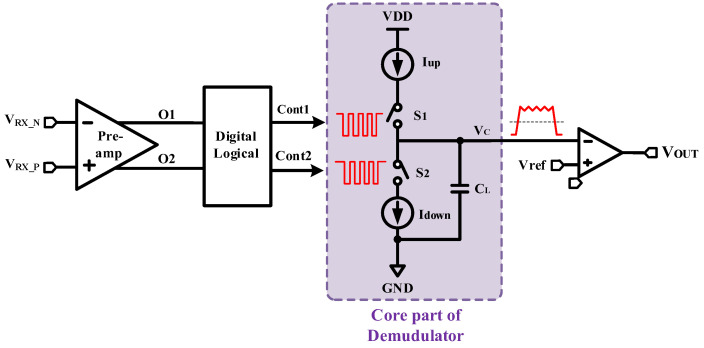
Basic principle of demodulator.

The challenge of the demodulation circuit is to maintain accuracy and efficiently restore the high-frequency output signal to the input signal while maintaining low delay. Figure 8 shows the block diagram of the overall demodulation circuit. The core of the demodulation circuit contains two current sources, two complementary MOS switches, and a 100 fF capacitor. The amplified differential signal is first input to the digital logic unit for signal processing, and obtains two in-phase non-interleaved clock signals as the result, to control the upper and lower MOSFET switches, respectively. Thus, the capacitors were subjected to irregular charging and discharging. The voltage of the upper pole plate of the capacitor is used as the primary demodulation signal, which is input to the negative terminal of the comparator to compare with the reference voltage to obtain the final demodulation signal.

To achieve better demodulation, certain restrictions on the current magnitude and switch signal are needed. Firstly, a larger charging current and a smaller discharging current are chosen. Then, the capacitor exhibits fast charging and slow discharging, and the primary demodulation signal can be pulled up quickly when the input signal jumps from low to high. Secondly, during the periodic switching process, a smaller leakage current ensures a slow decrease in signal and minimal amplitude during the opening period of the low-side current source. At the same time, a larger pull-up current continuously charges the capacitor during the opening period of the high-side current source, which can maintain a high level of output.

**Figure 9:**
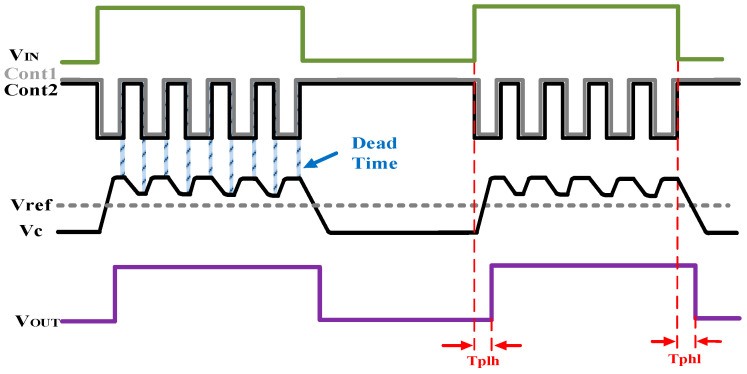
Diagram of DE operation waveforms.

On the other hand, a certain dead time should be set for the two control signals to prevent high-current throughput caused by simultaneous opening of the upper and lower MOS switches. Figure 9 shows the operation waveform of the core demodulation unit. The logical relationship between the upper and lower current source switches S_1_ and S_2_ and the input signal *V*_IN_ should be as follows: during the period when *V*_IN_ is low, S_1_ is turned off and S_2_ is turned on, and the output corresponds to a low level; during the period when *V*_IN_ is high, S_2_ is turned off, and then S_1_ is turned on again after a certain dead time, and so on, repeatedly, alternately. When the input level into the next cycle flipped to a low level, ensure S_2_ is open after S_1_ is off. Note that, due to the switch, the single turn-on time is shorter, and the size of the charge–discharge current will have a greater impact on the output waveforms, so it is better to select a higher-resolution current source, such as Cascode structure with high output impedance [20].

### 2.3. Driver

**Figure 10:**
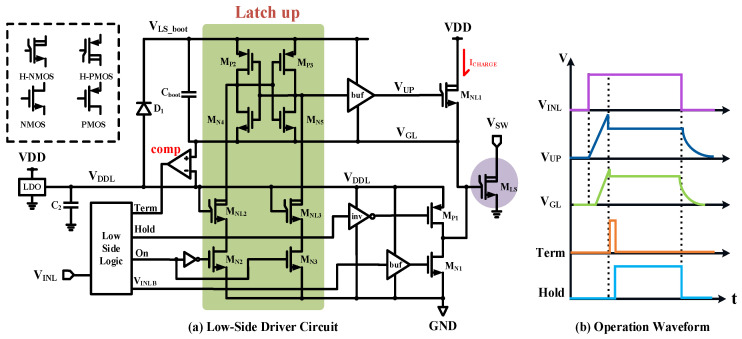
Proposed low-side driver.

Figure 10 shows the circuit diagram of the high-voltage low-side driver. The built-in LDO with capacitor C_2_ provides power for the low-voltage domain of the low-side drive. The larger-sized transistor M_NL1_ and M_P1_ work sequentially to provide the charging circuit for the power transistor M_LS_ during the turn-on process; M_N1_ with a buffer provides a discharging circuit for the M_LS_; M_N2_~M_N5_, M_NL2_~M_NL3_, and M_P2_~M_P3_ comprise the low-side level-shifter. Diode D_1_ and bootstrap capacitor C_boot_ are used to implement the lifting of the potential of the floating power supply rails; the comparator detects the gate voltage of M_LS_ to achieve the gate protection function.

There is a high-voltage level-shifter inside the low-side Driver. If the gate signal of the pull-up transistor M_NL1_ is supplied from the power rail *V*_DDL_, the maximum power device M_LS_ gate drive voltage can only reach *V*_DDL_-*V*th, which is just suitable for the low-voltage drive applications. To better drive the M_LS_, a level-shifting circuit utilizing bootstrap capacitor C_boot_ is designed in order to generate a floating power supply *V*_LS_boot_. When the low-side input *V*_INL_ is low, C_boot_ is charged by VDD through diode D_1_, and the voltage difference between the two ends of C_boot_ is VDD minus the conduction voltage drop of D_1_. When *V*_INL_ is flipped high, the gate voltage of M_LS_ is instantly lifted to VDDL; because the voltage across the capacitor cannot change abruptly, *V*_LS_boot_ will be lifted to 2 times VDDL, thus achieving the level-shifting circuit.

When the input to the low-side driver is low, the discharge current of M_LS_ flows directly into GND through the pull-down switch M_N1_. When the input is high, the M_LS_ turns on in two stages. Firstly, M_NL1_ conducts to charge the M_LS_ gate. In this stage, charging current comes from the Drive supply VDD instead of the floating supply rail *V*_DDL_, that can eliminate the high-current disturbances on the LDO output supply, thus avoiding the use of off-chip capacitors to maintain the power rails for the low-side drive. When M_LS_ gate voltage is higher than 5 V, the turn-on process turns to gate voltage holding stage. At this point, the output of the comparator is processed by the logic unit into a short-pulse Term to close the gate-charging process to protect the M_LS_ from breakdown. Meanwhile, M_P1_ is open in order to maintain the gate voltage of M_LS_ with a small pull-up current. The threshold voltage of the comparator should be designed slightly lower than the supply rail *V*_DDL_ for compensating the comparator’s transmission delay.

**Figure 11:**
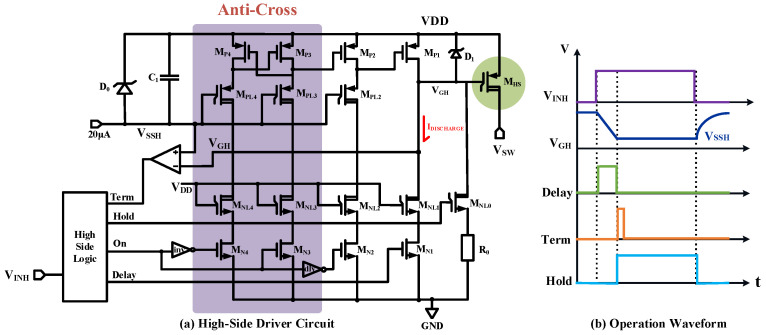
Proposed high-side driver.

The high-voltage high-side driver is shown as Figure 11, which includes a floating ground rail *V*_SSH_ generation circuit, level-shift circuit consisting of M_N2_ to M_N4_ branches with anti-cross conduction functionality, and a gate protection circuit for the power transistor. The sizes of M_P1_ that is responsible for pulling up M_HS_, and M_NL1_ that is pulling down M_HS_ are larger than other transistors. The bias voltage *V*_DDL_ of the high-voltage isolation transistors M_PL2_~M_PL4_ is generated by a 25 μA current source and voltage regulator D_0_. A bypass capacitor C_1_ is also included to compensate for charge losses caused by switching and stabilize the *V*_DDL_ potential.

When *V*_INH_ is low, the built-in level-shifter circuit converts the output signal of the low-voltage domain to the high-voltage domain (VDD~VDD-5V) range; furthermore, the pull-up switch M_P1_ can turn off the power transistor M_HS_. In the high-side level-shifter, the two branches of M_N3_ and M_N4_ trigger the high-side latch unit with short pulses to quickly lock the high-side output state. As the output stage, the M_N2_ branch aims to improve the driving capability of the level shifter. The gate drive signal of M_N2_ should be set with delay time to prevent M_P1_ and M_N1_ from conduction simultaneously, so that it can avoid generating a penetrating current flowing from power supply V_DD_ to ground.

When *V*_INH_ is high, large-sized M_N1_ pulls down the gate voltage to open M_HS_ quickly. As the gate drain current flows into the reference ground, spike voltage perturbation on the floating rail *V*_DDL_ will be eliminated. When the gate voltage of M_HS_ is lower than *V*_DDL_, the built-in high-voltage comparator immediately outputs a short-pulse Term signal to turn off M_N1_; in this way, the gate voltage is well-protected. At the same time, the Hold signal is switched to high to open the gate voltage holding branch at M_NL0_ to maintain the gate voltage of M_HS_ by using the reverse breakdown characteristic of the voltage regulator D_1_.

### 2.4. CMTI

CMOS CMTI refers to the irreversible effects of a transient signal in the reference ground on either side of the input or output side, that will be coupled as a common-mode transient pulse into the other side through the isolation capacitor [21]. CMT events with different rates of change produce common-mode transient pulses of different magnitudes. A common-mode event in TX will have two effects on the RX. Firstly, it will generate a large common-mode to differential-mode signal. Secondly, it will result in an offset of the DC operating point at the RX input; a big offset results in erroneous data transmission, such as outputting a high level when the transmitted data should be low.

**Figure 12:**
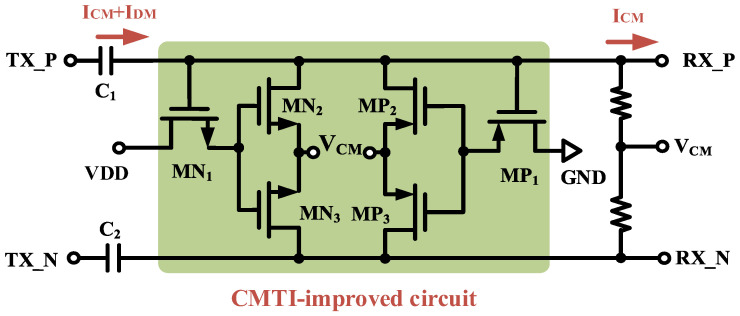
Active common-mode filter circuit.

To avoid this situation, a novel active common-mode filtering circuit is proposed, as shown in Figure 12. A small MOS device is utilized to reduce the offset of the DC operating point at the receiver side when a high-speed CMT event occurs. When no CMT event occurs, only the differential-mode small-signal current is output on the isolation capacitor, both M_N1_ and M_P1_ are in the offset, and the active common-mode filter circuit does not work. When a positive CMT event occurs, the transient pulse signal will cause the isolation capacitor to output a large positive transient signal; at this time, M_N1_ was opened, and the isolation capacitor output differential-mode current into the reference ground. Similarly, in case of a negative CMT event, it will cause the isolation capacitor to output a large negative transient signal to make M_P1_ open, and the compensation current is poured into the receiver from the power supply to maintain the stability of the common-mode potential at the receiver. Additionally, the DC operating point due to common-mode transients can be further reduced by decreasing the size of transistors M_N1_ and M_P1_ to lower their threshold voltages.

**Figure 13:**
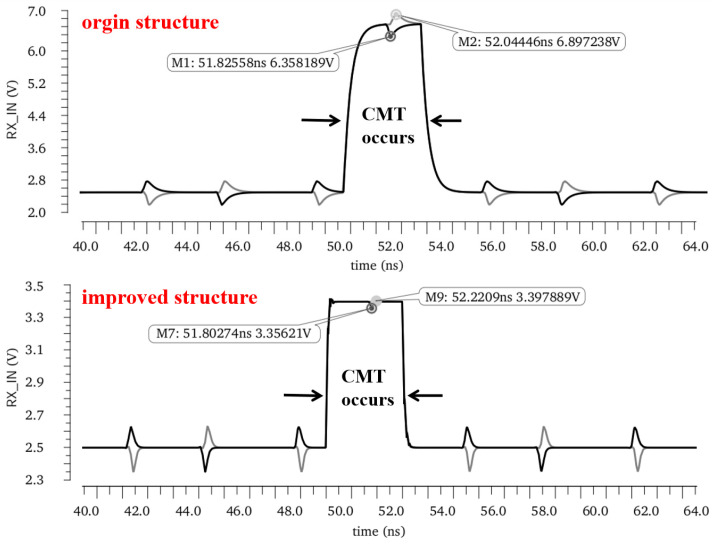
Diagram of waveform comparison with/without common-mode filter circuit.

The transient simulation results of the critical node are shown in Figure 13. When a CMT event of 150 kV/μs occurs on the TX side, gate breakdown will occur in the input device at the receiver side of the isolator where clamping is not added. Instead, the inclusion of an active common-mode filter circuit transmits the full differential signal by filtering the common-mode current. And it also avoids transmission errors due to DC operating point offset by reducing the transient voltage drop falling on the common-mode resistor.

## 3. Post-Simulation Results

**Figure 14:**
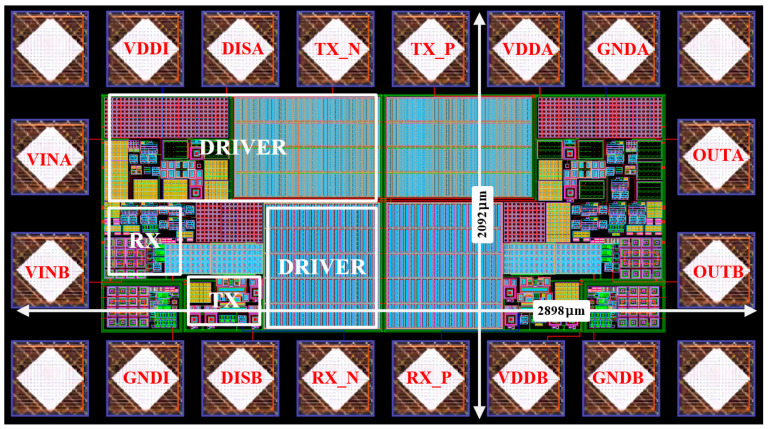
Layout of dual-channel capacitive digital isolation driver.

Figure 14 shows the overall layout of a dual-channel isolated driver. Based on the X-Fab 0.18 μm CMOS process, the overall layout size is 2092 × 2898 μm^2^. Every individual transmission channel consists of a TX, RX, and Driver. From the layout, it can be observed that the dual-channel layout is highly symmetric, which is favorable for improving the CMTI of the digital isolator. To make the post-simulation process closer to reality, some parasitic parameters in the isolation part that are easy to ignore are, additionally, considered. Firstly, parasitic inductance and resistance are added to the wires connecting the single channels between the back-to-back capacitors to simulate the actual bonding wires. The effect of the larger parasitic capacitance between the pole plate and the substrate under the isolation capacitor is also considered. In this way, the error between the post-simulation results and the experimental results is minimized.

**Figure 15:**
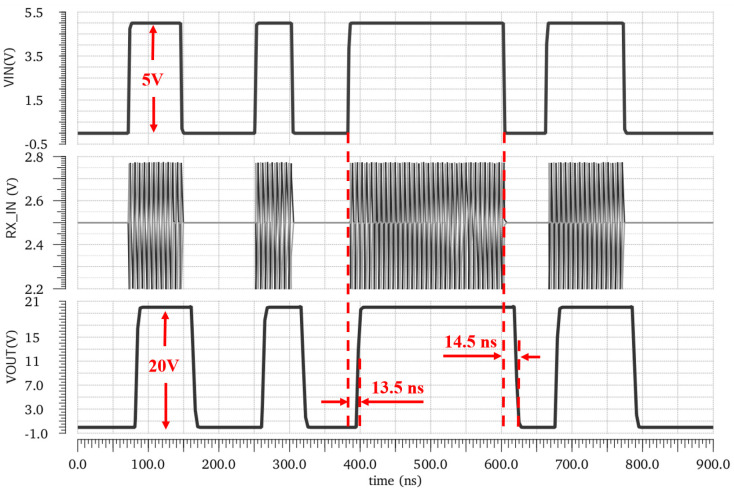
Simulated waveforms of isolator driver with 1.2 nF load.

**Figure 16:**
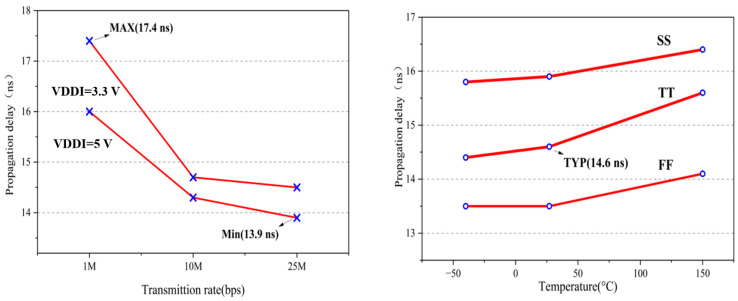
Propagation delay vs. supply voltage and temperature.

Figure 15 shows the simulated waveforms of the key nodes of the TX, RX, and Driver modules. The input is a five-bit pseudo-random bit sequence (PRBS) mode signal. The typical propagation delay from the input pin to the output pin is 15 ns at the TT process, at 27 °C, with a 1.2 nF load. The post-simulation results illustrate that the designed isolated driver achieves an accurate modulation–demodulation function and an efficient driving function at different data rates. The propagation delay is insensitive to changes in temperature and transmission rate as illustrated in Figure 16.

**Figure 17:**
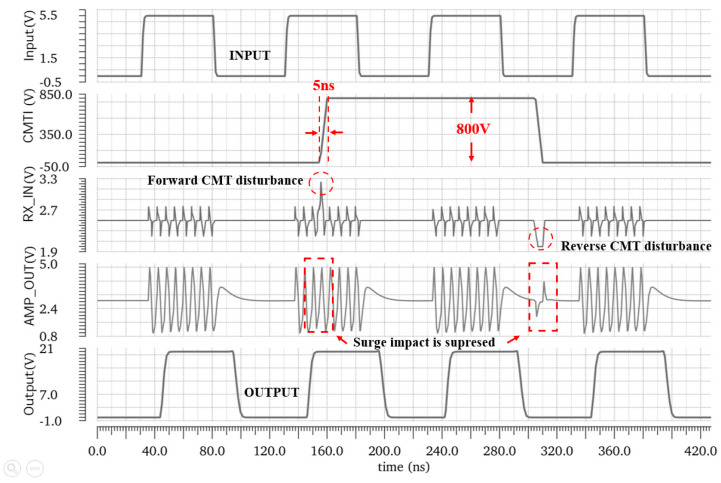
CMTI events.

Figure 17 shows the CMTI performance of the digital isolator. A high-voltage signal with an edge rate of 150 kV/μs is added between TX and RX to simulate CMT events. When a 10 MHz, 50% duty cycle square wave signal is input, the back results show that the output has no transmission error. Table 1 compares the simulation results of this design with other previous work. The drive capability of this work is 80 times that of [22], 50 times that of [13], and 25 times that of [12], respectively. The proposed isolated driver achieves a high CMTI capability and low propagation delay.

## 4. Conclusions

This paper proposes a dual-channel configurable capacitor digital isolation driver in the 0.18 μm CMOS process. A high data transmission rate and low propagation delay are achieved by using the OOK modulation technique and a fully differential transmission form. The fully integrated high-voltage level-shift driver without an off-chip capacitor has a strong driving capability for power devices. An active common-mode filter circuit is proposed to further improve the CMTI capability of the chip, which ultimately achieves a CMTI of higher than 150 kV/μs. The typical propagation delay is less than 15 ns at a 25 Mbps transmission rate with a 1.2 nF load. It can be configured as two signal-side drivers or a DT-programmable half-bridge driver. The isolated drivers can meet the high efficiency and reliability requirements of a wide variety of power supply applications.

## Figures and Tables

**Table 1:** Chip specifications.

Parameter	This Work	AD1100 [23]	MAX14850 [13]	TI7041 [12]	1EDI [24]
Isolation Element	Capacitor	Transformer	Transformer	Capacitor	Transformer
Max Data Rate	25 Mbps	25 Mbps	50 Mbps	2 Mbps	2 Mbps
CMR	4 kV	2.5 kV	0.6 kV	3 kV	1.2 kV
CMTI	160 kV/μs	35 kV/μs	100 kV/μs	100 kV/μs	100 kV/μs
Propagation Delay	15 ns	18 ns	18 ns	140 ns	120 ns
Output Peak Source/Sink Current	2 A/4 A	2.5 mA	4 mA	15 mA	2 A/4 A

## Data Availability

All the data are reported/cited in the paper.

## References

[1] Huang C.-Y., Shen W.C., Tseng Y.-H., King Y.-C., Lin C.-J. (2012). A contact-resistive random-access-memory-based true random number generator. IEEE Electron Device Lett..

[2] Balatti S., Ambrogio S., Wang Z., Ielmini D. (2015). True random number generation by variability of resistive switching in oxide-based devices. IEEE J. Emerg. Sel. Top. Circuits Syst..

[3] Altoobaji I., Hassan A., Ali M., Audet Y., Lakhssassi A. (2023). A Low-Power 0.68-Gbps Data Communication System for Capacitive Digital Isolator With 1.9-ns Propagation Delay. IEEE Trans. Very Large Scale Integr. (VLSI) Syst..

[4] Inohara M., Sugahara S. Basic Characteristics of Thin-Film Single-Layer Coreless Micro-Transformers for Digital Isolators. Proceedings of the 2022 International Power Electronics Conference (IPEC-Himeji 2022-ECCE Asia).

[5] Sadrimanesh H., Blaquière Y., Nabki F. Toward 2.5 D structures for multi-channel MEMS acoustic-based digital isolators using through silicon openings. Proceedings of the 2023 21st IEEE Interregional NEWCAS Conference (NEWCAS).

[6] Altoobaji I., Ali M., Hassan A., Audet Y., Lakhssassi A. A high speed fully integrated capacitive digital isolation system in 0.35 µm CMOS for industrial sensor interfaces. Proceedings of the 2021 19th IEEE International New Circuits and Systems Conference (NEWCAS).

[7] Ke J., Feng J., Liu C., Guan Z., Xu H., Zhao M. Digital Isolator Based on On-Chip Transformer for High-Voltage SiC MOSFET. Proceedings of the 2023 IEEE 7th Conference on Energy Internet and Energy System Integration (EI2).

[8] Motto E., Donlon J., Watabe K., Kazunari H., Araki T. A Monolithic 500V, 1A Three Phase Motor Driver with Small Line Surface Mount Package. Proceedings of the 2007 IEEE Industry Applications Annual Meeting.

[9] Kaeriyama S., Uchida S., Furumiya M., Okada M., Maeda T., Mizuno M. (2011). A 2.5 kV isolation 35 kV/us CMR 250 Mbps digital isolator in standard CMOS with a small transformer driving technique. IEEE J. Solid-State Circuits.

[10] Qu Y.D.C.L.L.S.X.Y.W. A Fully integrated, Low-Cost, High Channel Utilization Digital Isolator in Standard CMOS with 50Mbps and 100kV/μs CMTI. Proceedings of the Asia Conference on Power and Electrical Engineering.

[11] Nguyen V.H., Ly N., Alameh A.H., Blaquière Y., Cowan G. (2021). A versatile 200-V capacitor-coupled level shifter for fully floating multi-MHz gate drivers. IEEE Trans. Circuits Syst. II Express Briefs.

[12] Instruments T. ISO7041 Ultra-Low Power Four-Channel Digital Isolator. https://www.ti.com/lit/ds/symlink/iso7041.pdf?ts=1716176779603.

[13] MAXIM MAX14850. https://www.analog.com/media/en/technical-documentation/data-sheets/MAX14850.pdf.

[14] Xiong Z., Pan D., Li G., Cheng L. A 250Mbps 100kV/µs CMTI On-Chip Double-Isolated Transformer-Based Digital Isolator. Proceedings of the 2022 IEEE International Conference on Integrated Circuits, Technologies and Applications (ICTA).

[15] Luo Y., Fang J., Zhang E., Li M., Zhang B. A Novel Cross-Over CMR Transformer Technology for Magnetic Isolation Gate Driver Applications. Proceedings of the 2019 31st International Symposium on Power Semiconductor Devices and ICs (ISPSD).

[16] Ke X., Ma D.B. A 3-to-40V V IN 10-to-50MHz 12W isolated GaN driver with self-excited t dead minimizer achieving 0.2 ns/0.3 ns t dead, 7.9% minimum duty ratio and 50V/ns CMTI. Proceedings of the 2018 IEEE International Solid-State Circuits Conference-(ISSCC).

[17] Landau H. (1967). Sampling, data transmission, and the Nyquist rate. Proc. IEEE.

[18] Kelam M., Battu B.Y., Abbas Z. A Compact, Power Efficient, Self-Adaptive and PVT Invariant CMOS Relaxation Oscillator. Proceedings of the 2020 IEEE Computer Society Annual Symposium on VLSI (ISVLSI).

[19] Zangpo J., Póvoa R., Guilherme J., Horta N. An Integrated LC Oscillator with Self Compensation for Frequency Drift and PVT Corners Variations. Proceedings of the 2018 25th IEEE International Conference on Electronics, Circuits and Systems (ICECS).

[20] Chen G., Jin Z., Deng Y., He X., Qing X. (2017). Principle and topology synthesis of integrated single-input dual-output and dual-input single-output DC–DC converters. IEEE Trans. Ind. Electron..

[21] Xie H., Wang Z., Liu G., Lu J., Yi X. (2022). A novel active—Input cascode current mirror with high precision and low power dissipation. Eng. Rep..

[22] Ke X., Chen M. (2020). High Common-Mode Transient Immunity High Voltage Level Shifter.

[23] Devices A. iCoupler Digital Isolator ADuM1100. https://www.analog.com/media/en/technical-documentation/data-sheets/ADUM1100.pdf.

[24] Infineon 1EDI EiceDRIVER. https://www.infineon.com/dgdl/Infineon-1EDIXXI12AF-DataSheet-v01_10-EN.pdf?fileId=db3a3043427ac3e201428e648a333734.

